# Pharmacologic Profile of Naloxegol, a Peripherally Acting *µ*-Opioid Receptor Antagonist, for the Treatment of Opioid-Induced Constipation

**DOI:** 10.1124/jpet.116.239061

**Published:** 2017-05

**Authors:** Eike Floettmann, Khanh Bui, Mark Sostek, Kemal Payza, Michael Eldon

**Affiliations:** AstraZeneca UK Ltd., Cambridge, United Kingdom (E.F.); AstraZeneca Pharmaceuticals LP, Wilmington, Delaware (K.B.); AstraZeneca Pharmaceuticals LP, Gaithersburg, Maryland (M.S.); AstraZeneca Canada, Montreal, Quebec, Canada (K.P.); and Nektar Therapeutics, San Francisco, California, Primary laboratory of origin: AstraZeneca Pharmaceuticals LP, Wilmington, Delaware (M.E.)

## Abstract

Opioid-induced constipation (OIC) is a common side effect of opioid pharmacotherapy for the management of pain because opioid agonists bind to *µ*-opioid receptors in the enteric nervous system (ENS). Naloxegol, a polyethylene glycol derivative of naloxol, which is a derivative of naloxone and a peripherally acting *µ*-opioid receptor antagonist, targets the physiologic mechanisms that cause OIC. Pharmacologic measures of opioid activity and pharmacokinetic measures of central nervous system (CNS) penetration were employed to characterize the mechanism of action of naloxegol. At the human *µ*-opioid receptor in vitro, naloxegol was a potent inhibitor of binding (*K*_i_ = 7.42 nM) and a neutral competitive antagonist (p*A*_2_ - 7.95); agonist effects were <10% up to 30 *μ*M and identical to those of naloxone. The oral doses achieving 50% of the maximal effect in the rat for antagonism of morphine-induced inhibition of gastrointestinal transit and morphine-induced antinociception in the hot plate assay were 23.1 and 55.4 mg/kg for naloxegol and 0.69 and 1.14 mg/kg by for naloxone, respectively. In the human colon adenocarcinoma cell transport assay, naloxegol was a substrate for the P-glycoprotein transporter, with low apparent permeability in the apical to basolateral direction, and penetrated the CNS 15-fold slower than naloxone in a rat brain perfusion model. Naloxegol-derived radioactivity was poorly distributed throughout the rat CNS and was eliminated from most tissues within 24 hours. These findings corroborate phase 3 clinical studies demonstrating that naloxegol relieves OIC-associated symptoms in patients with chronic noncancer pain by antagonizing the *µ*-opioid receptor in the ENS while preserving CNS-mediated analgesia.

## Introduction

Opioid-induced constipation (OIC), characterized by decreased frequency of bowel movements, straining, hard stools, and incomplete evacuation (Bell et al., 2009a; Tuteja et al., 2010), is the most prevalent and bothersome side effect associated with opioid pharmacotherapy (Cook et al., 2008; Bell et al., 2009a). OIC results from the binding of opioid agonists to *µ*-opioid receptors in the enteric nervous system (ENS) (Pappagallo, 2001).

Opioid binding in the ENS affects gastrointestinal (GI) function by altering several GI mechanisms: inhibition of gastric motility and propulsion of the small and large intestine, diminished secretions throughout the digestive system, increased anal sphincter tone, impairment of anal sphincter reflexes associated with rectal distension, and increased absorption of water from bowel contents (Pappagallo, 2001). Conventional therapies for the treatment of constipation (e.g., dietary changes, stool softeners, and laxatives) do not target all of the underlying mechanisms of OIC and have therefore been associated with limited efficacy in this context (Koopmans-Klein et al., 2016; LoCasale et al., 2016). OIC was found to negatively affect pain management, productivity, and patients’ quality of life (Bell et al., 2009b) and to increase healthcare utilization and costs (Fernandes et al., 2016). The risk of having an all-cause in-patient hospitalization, emergency department visit, and office or other outpatient visit was nearly twice higher in patients on chronic opioid treatment with OIC (Fernandes et al., 2016).

**Fig. 1.**
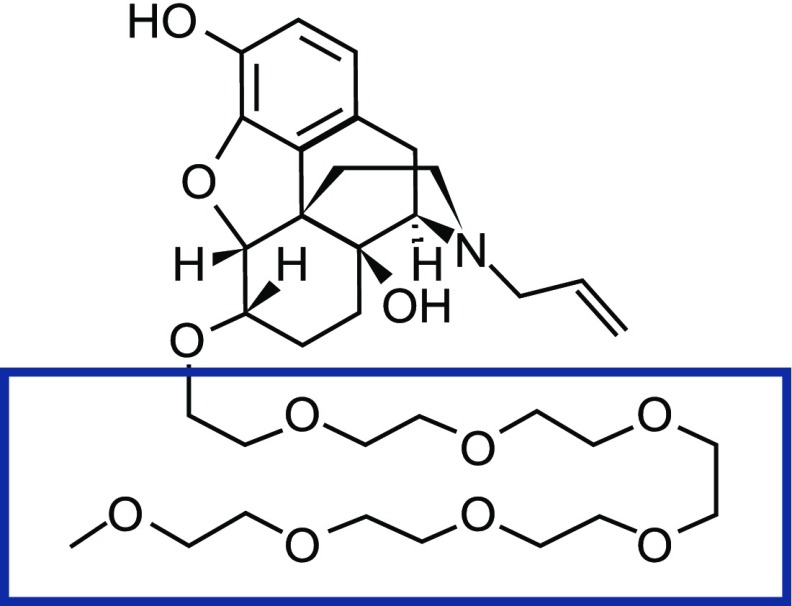
Chemical structure of naloxegol, a PEGylated derivative of naloxol, which is derived from naloxone. The box encompasses the low molecular weight polyethylene glycol (PEG) chain that is covalently bound to the morphinan ring-based structure of naloxone.

Naloxegol is a polyethylene glycol (PEG) derivative of naloxol, which is derived from naloxone that acts as a peripherally acting *µ*-opioid receptor antagonist (PAMORA) and constitutes a new treatment of OIC (Fig. 1) (Garnock-Jones, 2015). In 2014, the European Medicines Agency approved naloxegol (Moventig, AstraZeneca AB, Södertälje, Sweden) as treatment of OIC in adult patients who have had an inadequate response to laxatives and the US Food and Drug Administration approved naloxegol (Movantik, AstraZeneca Pharmaceuticals LP, Wilmington, DE) as treatment of OIC in adult patients with chronic noncancer pain.

The effectiveness of naloxegol was established in two identical Phase III, multicenter, randomized, double-blind, placebo-controlled trials conducted in 1352 patients who had been taking a stable dose of an oral opioid for noncancer pain. Patients were randomized to once daily treatment with naloxegol 12.5 or 25 mg or placebo. The primary end point was defined as the 12-week response rate (≥3 spontaneous bowel movements per week and an increase from baseline of ≥1 spontaneous bowel movements for ≥9 of 12 weeks and for ≥3 of the final 4 weeks) in the intention-to-treat population. Significantly more patients responded to naloxegol 25 mg than to placebo in both trials, and the response rate with the 12.5-mg dose was significantly higher than with placebo in only one trial (Chey et al., 2014).

This article describes the results of several studies that employed standard pharmacologic measures of opioid activity and pharmacokinetic measures of central nervous system (CNS) penetration, with an overall objective of characterizing the pharmacology and mechanism of action of naloxegol. Briefly, the objectives were to 1) establish the opioid receptor binding profile and functional activity of naloxegol in vitro, 2) determine the potency of naloxegol in vivo to antagonize the peripheral versus central effects of morphine, and 3) assess the ability of naloxegol to penetrate the blood-brain barrier (BBB) and distribute into various organs, including the brain and spinal cord.

## Materials and Methods

### Animal Care and Use

In situ rat brain perfusion experiments were conducted at accredited animal care facilities using approved protocols in accordance with standards in place at the times the studies were conducted. In vivo opioid receptor antagonism experiments and quantitative whole body autoradiography (QWBA) experiments were conducted in accordance with the relevant animal welfare laws in the United Kingdom.

### Peripheral Opioid Receptor Binding, Selectivity, and Functional Assays

#### Materials.

AstraZeneca (Mölndal, Sweden) provided the test compounds. Naloxegol was supplied as a resin synthesized by Nektar Therapeutics (batch number C537/1; San Francisco, CA). Methylnaltrexone bromide solution (Relistor, lot AGFH/13; Salix Pharmaceuticals, Bridgewater, NJ) was obtained from Mallinckrodt (Hazelwood, MO).

For the ligand binding studies, which were conducted at CEREP (Celle-Lévescault, France), the following materials were used: Tris and Tris-HCl (catalog number T1503; Sigma, France), MgCl_2_ (catalog number M9272; Sigma), EDTA (catalog number ED2SS; Sigma), NaCl (catalog number S9888; Sigma), [^3^H]diprenorphine (catalog number NET1121; Perkin Elmer, Belgium), [^3^H]d-Ala-d-Leu-enkephalin (catalog number NET648; Perkin Elmer), [^3^H]U69593 (catalog number NET952; Perkin Elmer), naltrexone (catalog number N3136; Sigma), and naloxone (catalog number N7758; Sigma).

For the guanosine 5-*O*-(3-[^35^S]thio)triphosphate (GTP*γ*S) binding experiments conducted at Eurofins Panlabs (Taipei, Taiwan), the following materials were used: HEPES (Sigma-Aldrich, St. Louis, MO), NaCl (Merck-Millipore, Darmstadt, Germany), MgCl_2_ (Sigma-Aldrich), dithiothreitol (DTT; Sigma-Aldrich), EDTA (Sigma-Aldrich), GDP (MP Biomedicals, Santa Ana, CA), scintillation proximity assay beads (WGA PVT beads, Perkin Elmer, Waltham, MA), [^35^S]GTP*γ*S (Perkin Elmer), morphine (TFDA, Taipei, Taiwan), [D-Pen^2^, D-Pen^5^]-enkephalin (DPDPE; Sigma-Aldrich), [D-Ala^2^, N-MePhe^4^, Gly-ol]-enkephalin (DAMGO, Sigma-Aldrich), and U69593 (Sigma-Aldrich).

#### Ligand Binding at and Selectivity for *µ*-, *δ*-, and *κ*-Opioid Receptors.

The in vitro affinity of naloxegol for cloned opioid receptor subtypes was determined and compared with that of the reference agent, methylnaltrexone. Competitive inhibition studies were performed on cloned opioid receptors expressed in membranes of Chinese hamster ovary (CHO) cells and conducted in triplicate (*n* = 3 independent experiments).

Radioligand binding assays were performed to evaluate the ability of these agents to inhibit specific radioligand binding to cloned opioid receptor subtypes. Half-maximal inhibitory concentration (IC_50_), equilibrium dissociation constant for the inhibitor (*K*_i_), and negative log of the equilibrium dissociation constant for the inhibitor (p*K*_i_) values were obtained. The radioligands and conditions for the binding studies are shown in Table 1. The receptor binding methods used for *µ* (Zhang et al., 1998)-, *δ* (Simonin et al., 1994)-, and *κ* (Meng et al., 1993)-opioid receptors have been described previously, with the exception that 100 mM NaCl was added to the incubation buffer in the *µ*-opioid receptor binding assay. All assays were conducted in 200-*µ*l volume. All assay incubations were terminated by filtration, and bound radioligand was quantitated by liquid scintillation spectrometry. The IC_50_ values were determined by nonlinear regression analysis of the competition curves generated with the mean of duplicate values using Hill equation curve fitting. The inhibition constants (*K*_i_) were calculated using the Cheng-Prusoff equation. The p*K*_i_ results from three independent experiments were analyzed for significant differences by one-way analysis of variance (ANOVA) followed by Bonferroni multiple comparison test.

**TABLE 1:** Details of opioid receptor binding studies

Receptor Assay	Membrane Source	Radioligand (Concentration)	*K*_d_	Nonspecific(Concentration)	Incubation Conditions	Incubation Buffer	Reference
*µ*	Human recombinant (HEK-293 cells)	[^3^H]diprenorphine(0.4 nM)	0.14 nM	Naltrexone (1 *μ*M)	120 min RT	50 mM Tris-HCl (pH 7.4), 100 mM NaCl, 5 mM MgCl_2_	Zhang et al., 1998
*δ*	Human recombinant (CHO cells)	[^3^H]DADLE(0.5 nM)	0.73 nM	Naltrexone (10 *μ*M)	120 min RT	50 mM Tris-HCl (pH 7.4), 5 mM MgCl_2_	Simonin et al., 1994
*κ*	Rat recombinant (CHO cells)	[^3^H]U69593(1 nM)	2 nM	Naloxone (10 *μ*M)	60 min RT	50 mM Tris-HCl (pH 7.4), 10 mM MgCl_2_, 1 mM EDTA	Meng et al., 1993

CHO, Chinese hamster ovary; DADLE, d-Ala-d-Leu-enkephalin; HEK, human embryonic kidney; *K*_d_, equilibrium dissociation constant of the radioligand; RT, room temperature; U69593, *N*-methyl-2-phenyl-*N*-(7-pyrrolidin-1-yl-1-oxaspiro[4.5]decan-8-yl)acetamide.

#### Functional Activity Assays.

Naloxegol was first tested for any *µ*-opioid agonist activity in [^35^S]GTP*γ*S filtration binding assays using membranes of human embryonic kidney (HEK)-293s cells stably expressing human *µ*-opioid receptors (maximum receptor density 0.29 pmol/mg protein). The experimental procedure was exactly as described previously (Payza, 2003). In brief, the assays were performed in 300 *µ*l in 96-well plates containing 10 *µ*g of the membranes, 50 mM HEPES, 20 mM NaOH, 100 mM NaCl, 1 mM EDTA, 5 mM MgCl_2_, 1 mM DTT, 15 *μ*M GDP, 0.4 nM [^35^S]GTP*γ*S, and 0.1% bovine serum albumin (pH 7.4). The plates were vortexed and incubated for 60 minutes at room temperature. After the incubation step, to remove the unbound [^35^S]GTP*γ*S, the plates were filtered and washed and bound [^35^S]GTP*γ*S was quantitated by scintillation spectrometry exactly as described previously (Payza, 2003). In each experiment, naloxone was included as a reference neutral antagonist, and DAMGO (10 *µ*M) was used as a reference agonist to define the maximum effect (E_max_) of the system.

Next, an assessment was carried out to determine whether naloxegol acts as a competitive *µ*-opioid antagonist. We evaluated the ability of naloxegol to elicit rightward shifts in the concentration-response curve of morphine in [^35^S]GTP*γ*S binding experiments. Human recombinant *µ*-opioid receptors were stably expressed in CHO-K1 cells (maximum receptor density 9.1 pmol/mg protein in membranes). In 96-well plates, incubation buffer (20 mM HEPES, pH 7.4, 100 mM NaCl, 10 mM MgCl_2_, 1 mM DTT, 1 mM EDTA), alone or containing various concentrations of morphine, was combined with the membranes (0.016 mg/ml), 3 *μ*M GDP, and scintillation proximity access beads. After 1 minute at room temperature, 0.3 nM [^35^S]GTP*γ*S was added in buffer alone or in buffer containing designated concentrations of naloxegol or methylnaltrexone, which was included as a reference antagonist in each experiment. DAMGO (10 *µ*M) was included as a reference agonist to define the E_max_ of the system.

After incubation for 30 minutes at 30°C, bound [^35^S]GTP*γ*S was quantitated by scintillation spectrometry. Raw data (obtained in counts per minute) were used in the subsequent curve-fitting analyses. Naloxegol and methylnaltrexone were tested in parallel in three independent experiments. An unpaired *t* test was used to compare the p*A*_2_ values of the two compounds. In the first data analysis method, raw data were fit by nonlinear regression to the Gaddum/Schild equation using Prism (GraphPad Software; San Diego, CA), with Schild slope and Hill slope fixed to 1.0, where

Y = [^35^S]GTP*γ*S binding (cpm);
X = concentration of morphine (log[molar]);
B = concentration of antagonist (molar);
EC_50_ = half-maximal effective concentration (10^log EC_50_);
Shift = 1 + (B/(10^[–1*p*A*_2_])^Schild slope;
LogEC = log (EC_50_*shift);
Y = bottom + (top – bottom)/(1 + 10^((logEC – X) * Hill slope)).

In the second method, to illustrate linearity and overall fit to the competitive interaction hypothesis, the EC_50_ values of morphine at each concentration of naloxegol and methylnaltrexone were obtained by fitting the raw data to the variable slope equation:


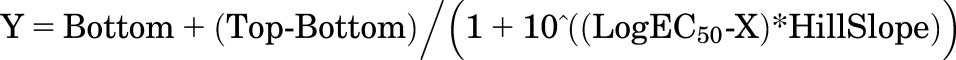


These values were used to calculate dose ratios and to generate a Schild plot with simple linear regression of data to the equation:


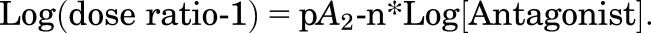


[^35^S]Guanosine 5-*O*-(3-[^35^S]thio)triphosphate binding experiments were also conducted with naloxegol (batch number 05-kspn588-14, Nektar Therapeutics, San Francisco, CA) in membranes of CHO cells expressing cloned human *δ*-opioid receptors and in membranes of HEK-293 cells expressing cloned human *κ*-opioid receptors. The effects of naloxegol in agonist mode of the assays were expressed relative to the maximal effect induced by DPDPE (10 *µ*M) at *δ*-opioid receptors and by U69593 (10 *µ*M) at *κ*-opioid receptors. In antagonist mode, naloxegol was tested for the ability to reverse agonism by DPDPE (300 nM) at *δ*-opioid receptors and U69593 (100 nM) at *κ*-opioid receptors. These were single experiments conducted across a range of 12 half-log concentrations, from 0.3 to 100 *μ*M, with 1 replicate per concentration; experimental conditions were as described for [^35^S]GTP*γ*S binding experiments with human recombinant *µ*-opioid receptors. Functional activity at *κ*-opioid receptors was also determined in an isolated tissue preparation from the rabbit vas deferens, using methods detailed previously (Oka et al., 1981).

### In Vivo Pharmacology of Naloxegol in Rat Models of Gastrointestinal Transit and Nociception

#### Materials.

Naloxegol (batch number LC-03N-114071) was synthesized by Nektar Therapeutics; stock solutions were stored at –20°C and protected from light. Morphine hydrochloride was supplied as a white crystalline powder (batch number 28305; Macfarlan Smith, Edinburgh, UK), and naloxone hydrochloride was supplied as a white solid (batch number 4A/48945; Tocris, Bristol, UK); both were stored at room temperature and protected from light.

#### Animals and Care.

Male Sprague-Dawley rats (approximately 6–8 weeks old at dosing, body weight approximately 150–260 g each) were supplied by Harlan UK Ltd. (Bicester, Oxon, UK). All animals were acclimated to the experimental unit for at least 3 days before the study. Rats tested in the GI transit assay were housed in grid-bottomed plastic cages (up to 4 animals per cage) suspended over paper-lined trays during the acclimation and on-study periods. Rats tested in the hot plate nociception assay were housed in solid-bottomed plastic cages with sawdust bedding (up to 5 animals per cage) during the acclimation and on-study periods. Holding and study areas were on an automatic 12-hour light:dark cycle (light hours: 0700−1900). The temperature set point was 20 ± 3°C; relative humidity was not controlled. Domestic mains tap water and a standard laboratory diet of known formulation (RM1[E] SQC; Special Diet Services, Witham, Essex, UK) were provided ad libitum.

#### Gastrointestinal Transit Model: Experimental Design.

The GI transit model was employed to determine the ability of naloxegol to antagonize morphine-induced constipation by measuring the distance traveled by a charcoal meal within the GI tract. Rats were arbitrarily allocated to treatment groups of 7 to 8 rats, as follows: intravenous 0.9% saline and oral 0.9% saline, intravenous morphine 10 mg/kg and oral 0.9% saline, and intravenous morphine 10 mg/kg with oral naloxegol (10, 30, 90 mg/kg) or naloxone (0.3, 1, 3, 10, 30 mg/kg). Naloxegol, naloxone, and morphine were formulated separately for administration in 0.9% saline. Naloxegol doses were prepared for oral administration in a volume of 10 ml/kg. Morphine doses were prepared for intravenous administration in a volume of 5 ml/kg.

Animals were fasted overnight before GI transit testing. Morphine or 0.9% saline was administered by tail vein injection at *t* = 0 minutes, followed by the administration of the saline vehicle, naloxone, or naloxegol by oral gavage at *t* = 5 minutes. Twenty-five minutes after treatment administration, 1 ml of a charcoal suspension was administered to each animal by oral gavage. Thirty minutes after charcoal ingestion, each rat was humanely euthanized by cervical dislocation and the intestine was exposed. The distance the charcoal had traveled along the intestine from the pyloric sphincter and the total intestinal length were measured. The distance the charcoal meal traveled in millimeters was calculated as a percentage of the total length of the intestine for each rat.

#### Gastrointestinal Transit Model: Statistics and Data Analysis.

Statistical comparisons were made between treatment groups using nonparametric (e.g., Kruskal-Wallis statistic, Dunn test, Mann-Whitney *U* test) statistical procedures. The decision to employ nonparametric tests was based on whether the groups being compared satisfied the homogeneity of variance criterion evaluated by the Levene mean test or *F* test. Nonparametric tests were used throughout to maintain continuity of assessment. The threshold for statistical significance was defined as *P*<0.05.

#### Nociception Model: Experimental Design.

The hot plate model of nociception was used to determine whether peripheral antagonism could be achieved at a dose that did not reverse the CNS-mediated analgesia induced by morphine. The time that an animal was able to tolerate heat from a hot plate indicated the degree of analgesia in effect. Rats were arbitrarily assigned to treatment groups of 8 to 10 rats per group, as follows: intravenous 0.9% saline and oral 0.9% saline, intravenous morphine 5 mg/kg and oral 0.9% saline, and intravenous morphine 10 mg/kg with oral naloxegol (10, 30, 90 mg/kg) or naloxone (1, 3, 10, 30 mg/kg).

To assess the dose range in which naloxone affected the antinociceptive properties of morphine, a dose range-finding test was conducted before the start of the main study. Four groups of adult male rats (*n* = 9) were dosed with intravenous morphine (5 mg/kg) and oral naloxone (3 mg/kg, *n* = 2; 10 mg/kg, *n* = 2; and 30 mg/kg, *n* = 3) or oral 0.9% saline (10 ml/kg, *n* = 2). These doses were used in the subsequent studies.

On each day of dosing, naloxegol, naloxone, and morphine were formulated separately for administration in 0.9% saline (batch numbers 02C11BE and 02J07G50; Baxter Healthcare Ltd., Compton, UK). All solutions were stored at room temperature and protected from light until use. Naloxegol and naloxone doses were prepared for oral administration in a volume of 10 ml/kg. Morphine doses were prepared for intravenous administration in a volume of 1 ml/kg.

Animals were fasted overnight before hot plate nociception testing. Animals were placed on a hot plate maintained at approximately 53°C. The withdrawal latency to heat exposure (withdrawal or shaking of hind feet, sharp withdrawal, or licking of forefeet, or attempting to escape by jumping) was recorded after the animal was removed from the hot plate. The maximum length of hot plate exposure was set at 15 seconds. A predose control response was measured before dosing to establish baseline withdrawal latencies. Predose baseline latencies were ranked, and the animals were allocated to treatment groups so that the mean baseline latencies were similar among groups. Morphine or saline was administered by tail vein injection at *t* = 0 minutes, followed by the administration of saline, naloxegol, or naloxone by oral gavage at *t* = 5 minutes. Each animal was submitted to the hot plate test at approximately 30 and 60 minutes after intravenous treatment administration.

#### Nociception Model: Statistics and Data Analysis.

Statistical comparisons were made between treatment groups using parametric (e.g., 1-way ANOVA, Dunnett *t* test, Student *t* test) or nonparametric (e.g., Kruskal-Wallis statistic, Dunn test, Mann-Whitney *U* test) statistical procedures. The decision to employ parametric or nonparametric tests was based on whether the groups being compared satisfied the homogeneity of variance criterion evaluated by the Levene mean test or *F* test.

Data from animals in treatment groups receiving intravenous morphine and oral naloxegol or naloxone were analyzed in comparison with animals receiving intravenous morphine and oral saline using the Levene mean test followed by an ANOVA and the Dunnett *t* test (naloxegol groups at predose and at 30 and 60 minutes postdose and naloxone groups at predose) or the Kruskal-Wallis and Dunn test (naloxone groups at 30 and 60 minutes postdose). Data from animals receiving intravenous morphine and oral saline were compared with data from animals receiving intravenous saline and oral saline using the *F* test followed by the Student *t* test (unpaired, 2-tailed). In all cases, the threshold for statistical significance was defined as *P*<0.05.

#### Dose-response Modeling for Gastrointestinal Transit and Nociception Assays.

In the GI transit assay, dose-response relationships were determined by calculation of the mean percentage of intestinal distance traveled for rats administered morphine and opioid antagonist as a fraction of the mean percentage of intestinal distance traveled for rats administered morphine and the saline vehicle. In the hot plate nociception assay, dose-response relationships were determined by calculation of the mean time spent on the hot plate for rats administered morphine and opioid antagonist as a fraction of the mean time on the hot plate for rats administered morphine and the saline vehicle. The doses achieving 50% of the maximal effect (ED_50_) generated for each opioid antagonist in the GI transit and hot plate assays were compared with determine whether peripheral antagonism could be achieved at a dose that did not reverse the CNS-mediated analgesia induced by morphine.

Data were fitted using the Origin (Version 7.5) Sigmoidal Fit tool, Dose-Response Fit. The logistical equation for fitting was (*A*_1_ – *A*_2_)/(1+(*x*/*x*_0_)*^p^*) + *A*_2_, where:

*x*_0_ = ED_50_;
*p* = power;
*A*_1_ = initial Y value;
*A*_2_ = final Y value.

The Y value at *x*_0_, also known as ED_50_, is halfway between the 2 limiting values *A*_1_ and *A*_2_: *y*(*x*_0_) = (*A*_1_ + *A*_2_)/2.

Analysis was also performed to demonstrate the separation between the ED_50_ values for naloxegol and naloxone.

### Human Colon Adenocarcinoma Cell Transport Assay

#### Materials.

Naloxone hydrochloride dihydrate (catalog number N7758; Sigma-Aldrich) was prepared as a 10 mM solution in dimethyl sulfoxide. Naloxegol (lot number RP01778-74-2) and elacridar were synthesized by Nektar Therapeutics; each reagent was prepared separately as a 10 mM solution in dimethyl sulfoxide. Cyclosporin A and verapamil were reagents provided by Absorption Systems, LC (Exton, PA), which conducted the study.

### Experimental Design

This assay measured the ability of naloxegol (and the centrally penetrant reference agent, naloxone) to permeate a bilayer of human colon adenocarcinoma (Caco2) cells to characterize its permeability and efflux properties. Caco2 monolayers were grown to confluence on collagen-coated, microporous, polycarbonate membranes in 12-well Costar Transwell plates (Corning Inc., Corning, NY). At the time the assays were conducted, the plates had been incubated for 21 to 28 days and cells were from passages 61 to 68. The permeability assay buffer for the donor chambers was Hanks’ balanced salt solution (pH 7.4, with 10 mM HEPES and 15 mM glucose). The buffer in the receiver chamber also contained 1% bovine serum albumin. The apparent permeabilities of naloxegol and naloxone were evaluated at fixed assay buffer concentrations of 10 *μ*M when studied alone and with verapamil and at 5 *μ*M when studied with cyclosporin A.

For inhibition experiments, all chambers were first preincubated for 30 minutes with assay buffer to presaturate any p-glycoprotein (P-gp) binding sites. After the preincubation period, the buffer was removed and replaced with fresh solution containing assay buffer and naloxegol or naloxone in the presence or absence of efflux transporter inhibitors (cyclosporin A, 10 *μ*M; verapamil, 100 *μ*M; or elacridar, 0.5 *μ*M). Cell monolayers were exposed to assay buffer on the apical side (A to B) or the basolateral side (B to A) and incubated at 37°C with 5% CO_2_ in a humidified incubator. Samples taken from the donor and receiver chambers at 2 hours were used to determine apparent permeability (P_app_). Each determination was performed in duplicate. After the individual monolayers were subjected to the test compounds, Lucifer Yellow flux was measured postexperiment to confirm the integrity of the monolayers.

#### Analysis of Membrane Permeability.

Concentrations of test molecules were measured using liquid chromatography-tandem mass spectrometry. Samples (10 *µ*l) were injected directly into a Hypersil BDS C18 high-pressure liquid chromatography (HPLC) column (30 × 2.1 mm inner diameter, 3-*µ*m particle size, with guard column; Thermo Fisher Scientific, Grand Island, NY) and a tandem mass spectrometer (PE SCIEX API2000 or API3000; AB SCIEX LLC, Framingham, MA). The mobile phase was 25 mM ammonium formate buffer, pH 3.5. Compounds were eluted with a linear gradient at a flow rate of 300 *µ*l/min. Eluted compounds were ionized using an electrospray interface.

Apparent permeability was measured in both absorptive (apical to basolateral; A to B) and secretory (basolateral to apical; B to A) directions. The efflux ratio for each molecule was calculated as P_app_ B to A/P_app_ A to B. The P_app_ and percent recovery were calculated as P_app_ = (*d*C_r_/*d*t) × V_r_/(A × C_A_) and percent recovery = 100 × [(V_r_ × C_r_^final^) + (V_d_ × C_d_^final^)]/(V_d_ × C_N_), respectively, where

*d*C_r_/*d*t is the slope of the cumulative concentration in the receiver compartment versus time in micromoles per second;
V_r_ is the volume of the receiver compartment in cubed centimeters;
V_d_ is the volume of the donor compartment in cubed centimeters;
A is the area of the cell monolayer (1.13 cm^2^ for 12-well Transwell);
C_A_ is the average of the nominal dosing concentration and the measured concentration in the donor chamber at 2 hours in micromoles;
C_N_ is the nominal concentration of the dosing solution in micromoles;
C_r_^final^ is the cumulative receiver concentration in micromoles at the end of the incubation period;
C_d_^final^ is the concentration of the donor in micromoles at the end of the incubation period.

### In Situ Rat Brain Perfusion Model

#### Materials and Equipment.

Naloxone hydrochloride dihydrate (reference compound; lot number 111K1379) was obtained from Sigma-Aldrich. Atenolol and antipyrine (phenazone) were reagents provided by Absorption Systems, who conducted the study. Naloxegol (lot number LC-03N-92117) was synthesized by Nektar Therapeutics.

#### Animals and Care.

Adult male Sprague-Dawley rats (approximately 11–12 weeks old and body weight approximately 300–400 g at arrival) were supplied by Hilltop Laboratory Animals, Inc. (Scottdale, PA). All animals were randomly assigned to treatment groups upon arrival and were acclimated to the experimental unit for at least 1 day before the study. Each animal was in the weight range of 308 to 410 g on the day of dosing.

Rats were housed in wire cages (2 animals per cage) during the acclimation and on-study periods. Before study initiation, water and a standard laboratory diet were provided for all animals ad libitum. Animals were not fasted before study initiation.

#### Experimental Design.

The rat in situ brain perfusion assay established the rate of permeation of naloxegol in comparison with that of naloxone.

##### Animal disposition.

Rats (*n* = 3/compound) were perfused with naloxone or naloxegol; data from 20 rats perfused solely with atenolol and antipyrine (part of an investigation of a large series of compounds) were used for control comparisons. All animals were euthanized by CO_2_ narcosis at the conclusion of the study or during the study if necessary.

##### Perfusion solution (perfusate) preparation.

Each test perfusate contained a test compound (target concentration, 20 *μ*M), atenolol (target concentration, 50 *μ*M), and antipyrine (target concentration, 5 *μ*M) dissolved in Krebs-Ringer solution (buffered with sodium bicarbonate, pH 7.4). Control perfusate lacked the test compound. To ensure the integrity of the perfusion preparation during the evaluation of test compounds, atenolol and antipyrine were included as permeation standards representing low and high BBB permeability, respectively.

Atenolol served as a low-permeability control; only values <10 pmol/g brain/s (perfused at 50 *μ*M) were considered acceptable. Because antipyrine is a lipophilic compound that easily crosses the BBB, deviation in the antipyrine value in a given perfusion preparation may suggest a failure in the surgical preparation of the brain. Uptake rates for the permeation standards atenolol and antipyrine were also determined in the absence of test compound.

##### Brain perfusion technique.

Perfusion was performed using the single time point method. Perfusate was infused into rats via the left external carotid artery at 20 ml/min using an infusion pump. This flow rate was selected to completely replace blood flow to the brain while maintaining normal physiologic pressure (80–120 mmHg). The perfusion duration was 30 seconds, followed immediately by perfusion for an additional 30 seconds with drug-free perfusate to remove any residual drug from the vasculature.

##### Sample preparation.

After perfusion, each rat was euthanized, the brain immediately removed from the skull, and the left cerebral hemisphere weighed. Each rat brain was added to 4 ml 20% methanol and homogenized using a Polytron homogenizer (ThermoFisher Scientific). The total volume of homogenate was measured and recorded.

Each sample was prepared and analyzed in triplicate: three aliquots were taken from each brain homogenate sample and treated independently to determine the drug concentration. For each aliquot, 200 *µ*l homogenate was diluted with 400 *µ*l acetonitrile containing internal standard and centrifuged for 10 minutes at 12,000 rpm. The supernatant (530 *µ*l) was placed into a clean centrifuge tube, and the samples were dried at 37°C under a stream of nitrogen. Dried samples were reconstituted with 125 *µ*l of 10% acetonitrile/90% water and again centrifuged for 10 minutes at 12,000 rpm. After centrifugation, 120 *µ*l of supernatant was placed into an HPLC vial pending analysis, as described below.

##### Quantification and analysis of drug concentrations (liquid chromatography-tandem mass spectrometry).

Reconstituted supernatants (10 *µ*l) were injected into a Capcell Pak C8 HPLC column (50 × 2.1 mm inner diameter; Phenomenex Inc., Torrance, CA) at 45°C or a Hypersil BDS C18 column (30 × 2.1 mm inner diameter, 3 *µ*m particle size; Thermo Fisher Scientific), each coupled to a tandem mass spectrometer (API4000; AB SCIEX LLC). The mobile phase was 25 mM ammonium formate buffer, pH 3.5. Compounds were eluted with a linear gradient at a flow rate of 250 *µ*l/min (Capcell Pak C8 column) or 300 *µ*l/min (Hypersil BDS C18 column). Eluted compounds were ionized using an electrospray interface.

Drug concentrations in brain homogenate were quantified against calibration curves generated by spiking the drugs into drug-free brain homogenate. Average values for the triplicate measurements were calculated and considered as *n* = 1. Summary statistics were calculated using Microsoft Office Excel, 2003 (Microsoft, Redmond, WA).

### Quantitative Whole Body Autoradiography

Quantitative whole body autoradiography was conducted to track the overall distribution of a single oral dose of radiolabeled naloxegol in adult male and pregnant female rats.

#### Materials and Equipment.

Naloxegol (batch number 1005, AstraZeneca, Wilmington, DE) was stored at 2° to 8°C and protected from light. ^14^C-radiolabeled naloxegol [1.0 mCi/ml; specific activity 49.5 mCi/mmol (2.80 MBq/mg); Isotope Chemistry, AstraZeneca] was stored at –20°C in the dark. Aquasafe 500 Plus liquid scintillation fluid was obtained from Zinsser Analytic (Maidenhead, UK). Sterile water was obtained from Hameln Pharmaceuticals (Gloucester, UK). A Fuji FLA 5000 Image Analyzer (pixel size 50 *μ*m^2^) was used (Fuji Photo Film; Tokyo, Japan).

#### Animals and Care.

Seven male Lister hooded rats (8–9 weeks old at dosing, body weight 255–290 g each) and 5 time-mated female Sprague-Dawley rats (body weight 347–392 g each) were supplied by Charles River UK Limited (Margate, Kent, UK). All animals were acclimated to the experimental unit before the study (males, 6 days; females, 13 days).

Male rats were group housed in solid-floored polycarbonate and stainless steel caging during the prestudy period. During the study period, they were multiply housed, where possible, in polycarbonate and stainless steel cages with raised wire mesh floors. Time-mated female rats were held in solid-bottomed cages with bedding and nesting material during the acclimation and on-study periods.

Animals were provided domestic mains tap water and a standard laboratory diet of known formulation (SDS Rat and Mouse Maintenance Diet No. 1 for male rats and No. 3 for time-mated female rats; Special Diet Services, Witham, Essex, UK) ad libitum. Holding and study areas were on an automatic 12-hour light:dark cycle (light hours: 0700−1900). Temperature and humidity measured during the study ranged from 19° to 22°C and from 43 to 58%, respectively. All rats were offered environmental enrichment during the acclimation period.

#### Dose Formulation, Specific Activity, and Dose Assessment.

The dose formulation was prepared on the morning of administration. Naloxegol (288.0 mg) was weighed into an amber glass container. Sterile water (21 ml) was added and the formulation was stirred for 3 minutes. [^14^C]naloxegol (0.81 ml) was added and the formulation was stirred for 4 minutes. After adding 8.19 ml sterile water (final volume, 30 ml; target concentration, 10 mg/ml), the formulation was stirred for 3 minutes. Care was taken to ensure that solutes were completely dissolved at each step to form a homogenous solution.

Before use, the homogeneity and radioactive concentration of [^14^C]naloxegol were confirmed by liquid scintillation counting analysis (6 × 10 *μ*l aliquots). Duplicate weighed amounts of the dose formulation (calculated from differences in syringe weights before and after dispensing) were dispensed into volumetric flasks (×6) and made up to volume with water.

The amount of [^14^C]naloxegol in the dose formulation was determined by calculating the mean radioactive content from liquid scintillation counting analysis. The concentration of the dose formulation was subsequently calculated from the determined amounts of radiolabeled and nonradiolabeled naloxegol dispensed and the final weight of the dose formulation.

#### Dose Administration and Determination.

Animals were fasted overnight before dose administration. Radiolabeled naloxegol was administered by oral gavage (target dose volume, 5 ml/kg) to achieve a target dose level of 50 mg/kg (5.55 MBq/kg). The dose received by each animal was determined with reference to the radioactive concentration, the weight of the dose administered, and the specific activity of [^14^C]naloxegol in the dose formulation. Male rats were administered a mean (±S.D.) of 5.62 (±0.06) MBq/kg; female rats received a mean of 5.60 (±0.03) MBq/kg.

#### Sample Preparation and Storage.

One male rat was humanely euthanized by CO_2_ narcosis at 0.5, 1, 4, 24, 48, 168, and 504 hours postdose, respectively. One time-mated female rat was humanely euthanized at 0.5, 1, 4, 24, and 48 hours postdose, respectively.

After removal of the whiskers, legs, and tails, the carcasses were frozen by immersion in a mixture of solid CO_2_ in hexane for approximately 30 minutes and then embedded in a block of carboxymethylcellulose, which was frozen in the same way. After equilibration at approximately –20°C, sagittal sections (30 *μ*m thick) were taken using a whole body cryomicrotome (Leica Biosystems GmbH, Nussloch, Germany). The sections were freeze dried in a chamber of the cryomicrotome before storage at ambient temperature. Carcasses were stored frozen (–20°C) until analysis. After analysis, samples were returned to storage at –20°C. Freeze-dried whole body sections were stored at room temperature.

#### Analysis of Total Radioactivity.

The radioactivity present in various organs and tissues in whole body sections was determined by QWBA. After exposure, the imaging plates were scanned using a phosphorimage analyzer. Tissues and organs of interest were quantified using AIDA image analysis software (version 4.06; Raytest Isotopenmeßgeräte GmbH, Straubenhardt, Germany). For analysis, representative whole body sections were placed into close contact with phosphor screens and left for a 7-day period. A set of external standards were also exposed on each phosphor screen. These standards were prepared from blood spiked with a serial dilution of a ^14^C-labeled reference solution, which was dispensed into holes drilled into a block of carboxymethylcellulose, frozen, and then sectioned in the same way as the animal samples.

After the phosphor screen was scanned, an image of the radioactivity in the sample was stored digitally. For quantitative analysis, six background areas were defined on each stored phosphor screen image. The software automatically calculated the mean background and subsequently subtracted this from all standards and tissues analyzed. A regression coefficient was derived by comparing the response of each standard with the nominal disintegrations per minute per gram over the range of radioactive concentration used and forcing the response curve through the origin. The concentrations of the standards used were in the range of 0.09 to 826.31 *μ*g Eq/g. The response curve was linear over these concentrations and was assumed to be linear to the limit of reliable determination.

Organs or tissues from representative autoradiograms were identified and integrated. The back-calculated tissue concentrations (*μ*g Eq/g) were then determined by the software. The limit of reliable measurement for each storage screen was calculated from the assessment of the mean background of the plate and taken to be three times the S.D. of the mean above background. At the specific activity used in this study, the limit of reliable measurement was in the range of 0.06 to 0.16 *μ*g Eq/g.

## Results

### Binding, Selectivity, and Functional Profile of Naloxegol In Vitro

In vitro competitive binding assays were conducted three times on three different days. Each experiment tested a range of concentrations of naloxegol and methylnaltrexone, with each concentration in triplicate. The results showed that naloxegol inhibited binding to all three opioid receptor subtypes. Mean ± S.E.M. p*K*_i_ values are summarized in Table 2. The *K*_i_ values of naloxegol at the cloned human *µ*-opioid receptor ranged from 6.5 to 8.5 nM. The p*K*_i_ values of naloxegol and methylnaltrexone corresponded to respective geometric mean *K*_i_ values of 7.42 nM and 22.1 nM, showing that naloxegol bound human *µ*-opioid receptors with threefold greater affinity than methylnaltrexone (*P* < 0.05). At cloned human *δ*-opioid receptors, the p*K*_i_ values of naloxegol and methylnaltrexone corresponded to respective geometric mean *K*_i_ values of 203 nM and 1.9 *μ*M (indicating that naloxegol bound such receptors with 9.4-fold greater affinity than methylnaltrexone, *P* < 0.001). At cloned rat *κ*-opioid receptors, the p*K*_i_ values of naloxegol and methylnaltrexone corresponded to respective geometric mean *K*_i_ values of 8.65 nM and 10.9 nM (*P* > 0.05). Apart from inhibition at opioid receptors, naloxegol did not significantly inhibit binding by >30% when screened across a panel of more than 300 receptors, ion channels, transporters, and enzymes at a final concentration of 10 *µ*M (data not shown).

**TABLE 2:** Binding affinity of naloxegol to opioid receptor subtypes Data are mean ± S.E.M. values from 3 independent experiments.

Receptor Subtype	Naloxegol p*K*_i_	Methylnaltrexone p*K*_i_	*P* Value*^a^*
*µ* (human)	8.13 ± 0.06	7.66 ± 0.08	<0.05
*δ* (human)	6.69 ± 0.05	5.72 ± 0.35	<0.001
*κ* (rat)	8.06 ± 0.05	7.96 ± 0.17	N.S.

ANOVA, analysis of variance; p*K*_i_, negative logarithm of the equilibrium dissociation constant for the inhibitor; N.S., not significant.

^a^ Comparison of p*K*_i_ values by 1-way ANOVA followed by Bonferroni multiple comparison test.

**Fig. 2.**
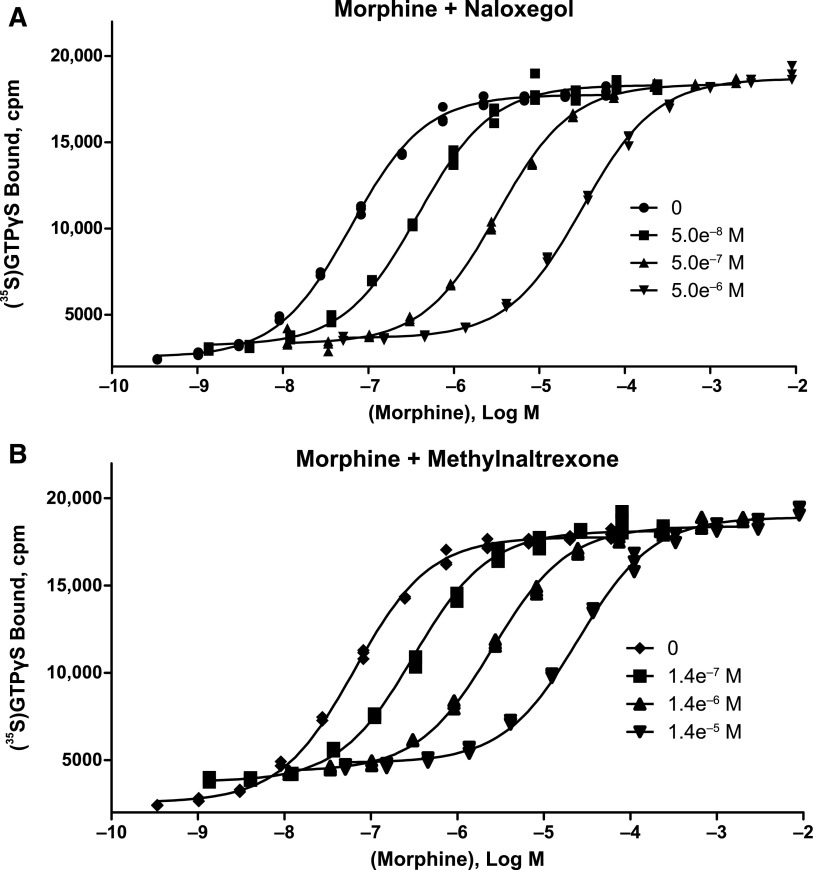
Competitive antagonism of naloxegol at the human *µ*-opioid receptor. The effects of 3 different concentrations of naloxegol (A) and methylnaltrexone (B) on morphine agonist concentration-response curves, as measured by guanosine 5-*O*-(3-[^35^S]thio)triphosphate ([^35^S]GTP*γ*S binding) (1 of 3 independent experiments) are shown. Each compound elicited a rightward shift in the morphine concentration-response curve with no reduction in E_max_.

**Fig. 3.**
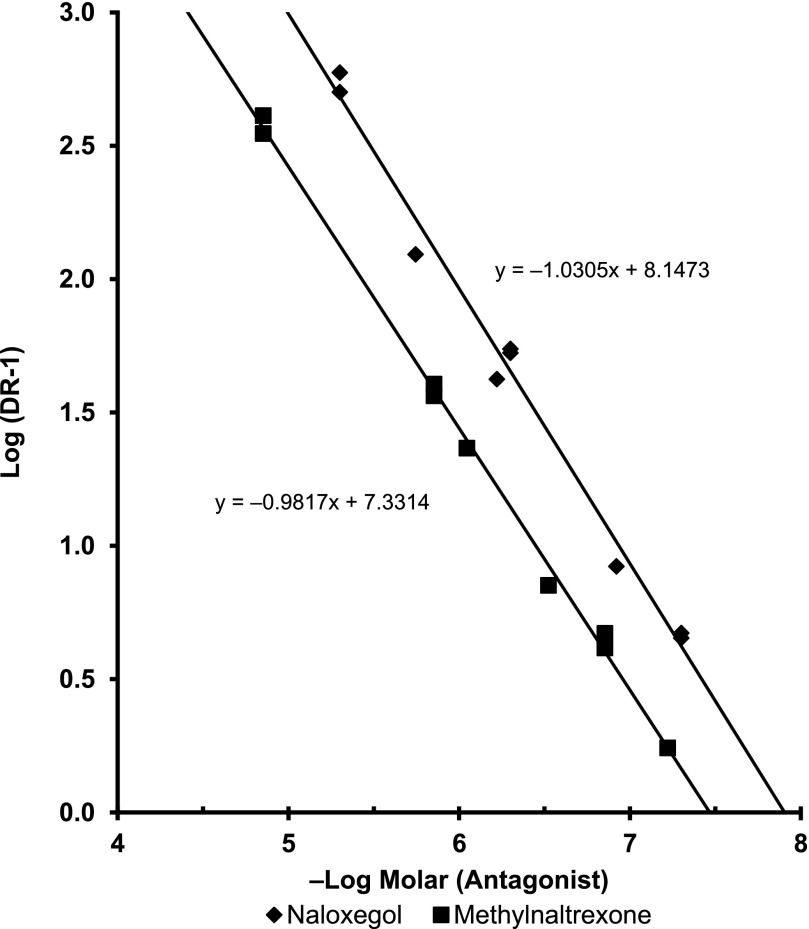
Naloxegol is a competitive neutral antagonist of morphine at the human *µ*-opioid receptor. This Schild plot depicts the effects of naloxegol (♦) and methylnaltrexone (▪) on morphine agonist concentration-response curves at the human *µ*-opioid receptor, as measured by [^35^S]GTP*γ*S binding (pooled results of 3 independent experiments for each compound). Note the respective Schild slopes: –1.03 for naloxegol and –0.982 for methylnaltrexone. DR, dose ratio.

Naloxegol was a potent and competitive neutral antagonist of morphine at the human *µ*-opioid receptor. In [^35^S]GTP*γ*S filtration binding assays using membranes of HEK-293 cells stably expressing human *µ*-opioid receptors, naloxegol (30 *μ*M) and naloxone (30 *μ*M) elicited 5.1 ± 2.2% and 6.3 ± 1.8% agonism, respectively, relative to DAMGO (mean ± S.D. of 8 values from 4 independent experiments). In Schild-type experiments (*n* = 3 independent experiments conducted in triplicate), naloxegol (Fig. 2A) and methylnaltrexone (Fig. 2B) each elicited parallel rightward shifts in the morphine dose-response curve without any accompanying reduction in E_max_, the maximal response produced by morphine. The results for both compounds fitted closely to the competitive antagonism model (*R ^2^*= 0.997−0.998), with Schild slopes of –1.03 for naloxegol and –0.982 for methylnaltrexone (Fig. 3).

Parallel testing of naloxegol and methylnaltrexone in three independent experiments yielded p*A*_2_ values of 7.95 ± 0.11 and 7.43 ± 0.02, respectively. These values correspond to respective equilibrium dissociation constant for the inhibitor in a functional assay (*K*_B_) values of 11 and 37 nM. Thus, consistent with the receptor binding data, naloxegol was 3.4-fold more potent than methylnaltrexone as an antagonist at human *µ*-opioid receptors (*P* = 0.0012, p*A*_2_ comparison, unpaired *t* test). Plate control wells in the Schild-type experiments also contained naloxegol and methylnaltrexone in the absence of morphine. At the highest concentrations tested, naloxegol (5 *µ*M) had <10% agonism (7.8 ± 1.3%) and methylnaltrexone (14 *µ*M) had 14.4±1.3% partial agonism relative to DAMGO (mean ± S.D. of 8 values from 2 independent experiments).

At the human *δ*-opioid receptor, naloxegol showed no agonism and weak antagonism, with an IC_50_ value of 866 nM in the [^35^S]GTP*γ*S functional assay. In the human *κ*-opioid receptor [^35^S]GTP*γ*S functional assay, naloxegol exhibited partial agonism. Naloxegol alone produced a concentration-dependent increase in binding, with an EC_50_ value of 47 nM and a maximal effect of 39% agonism relative to the reference *κ*-opioid agonist U69593. Data are from one experiment conducted in singlicate. Naloxegol also inhibited the response to U69593, with an IC_50_ value of 37 nM and maximal inhibition of 62% at 100 *µ*M. Functional studies in the rabbit vas deferens assay, however, indicated that naloxegol elicited no agonist activity at *κ*-opioid receptors at concentrations up to 10 *µ*M.

### Effects of Naloxegol on the Peripheral Nervous System- and Central Nervous System-Mediated Effects of Morphine In Vivo

Oral naloxone at doses ≥10 mg/kg and oral naloxegol at 90 mg/kg completely reversed the effect of morphine in the GI transit assay. Based on comparison of ED_50_ values (the dose achieving 50% of the maximal effect) derived from the dose-response modeling, naloxegol was approximately 33 times less potent than naloxone at antagonizing morphine in the GI tract (ED_50_ values of 23.1 and 0.69 mg/kg, respectively).

Oral naloxone at doses ≥10 mg/kg completely reversed the analgesic effect of morphine in the hot plate assay, with an ED_50_ of 1.14 mg/kg. The corresponding ED_50_ of naloxegol was 55.4 mg/kg, indicating that naloxegol was approximately 49-fold less potent than naloxone at antagonizing morphine analgesia.

The twofold separation in the peripheral nervous system (PNS) versus CNS effect observed in the rat was lower than that observed in humans (Eldon et al., 2015). However, this lower separation is most likely related to the greater extent of metabolism in the rat (2013 AstraZeneca data on file), resulting in greater exposure to potentially active metabolites with greater ability to penetrate the BBB than would be expected in humans.

### PEGylation Reduces the Passive Membrane Permeability of Naloxegol In Vitro and Makes Naloxegol Susceptible to Active Efflux

The respective permeabilities and efflux ratios of 10 *μ*M naloxegol in the presence of several P-gp inhibitors are summarized in Table 3. The apparent permeabilities of 10 *μ*M naloxone in the A to B and B to A directions were unaltered in the presence of cyclosporin A, elacridar, and verapamil. These data indicate that at a concentration of 10 *μ*M, naloxone transport across Caco2 monolayers is primarily passive. In contrast, the efflux ratio of naloxegol was reduced from 15 to 1, 1.3, and 1.1 in the presence of cyclosporin A, verapamil, and elacridar, respectively. These results suggest that naloxegol is a substrate for at least one apically directed efflux transporter, likely the P-gp transporter. Naloxegol also displayed low apparent permeability in both the A to B and B to A directions compared with naloxone, suggesting that the addition of the PEG chain to naloxegol reduces its passive permeability.

**TABLE 3:** Apparent permeability and efflux of naloxegol in the presence and absence of P-gp inhibitors, demonstrated by the Caco2 transport assay

Drug*^a^*	P-gp Inhibitor	P_app_ A→B(10^−6^ cm/s)	P_app_ B→A(10^−6^ cm/s)	Efflux Ratio
Naloxegol*^b^*	—	0.7	8.4	12.0
Naloxegol*^c^*	Cyclosporin A, 10 *μ*M	1.8	1.8	1.0
Naloxegol*^c^*	Elacridar, 0.5 *μ*M	2.3	2.5	1.1
Naloxegol*^c^*	Verapamil, 100 *μ*M	1.3	1.7	1.3
Naloxone*^b^*	—	27.3	25.0	0.9
Naloxone*^c^*	Cyclosporin A, 10 *μ*M	28.4	23.7	0.8
Naloxone*^c^*	Elacridar, 0.5 *μ*M	24.8	27.3	1.1
Naloxone*^c^*	Verapamil, 100 *μ*M	24.5	24.9	1.0

Caco2, human colon adenocarcinoma cell line; P_app_, apparent permeability; P-gp, P-glycoprotein.

^a^ Naloxegol and naloxone were tested at 10 *μ*M concentration in all experiments.

^b^ P_app_ and efflux ratio data are the arithmetic means of 3 separate experiments, each conducted in duplicate.

^c^ P_app_ data shown are the arithmetic means of respective single experiments conducted in duplicate.

The P-gp inhibitors employed in the in vitro Caco2 permeability assays inhibit multiple transporters in addition to the P-gp transporter. Cyclosporin A is an inhibitor of P-gp, multidrug resistance-associated protein 2, and the organic anion transporter protein-C; elacridar inhibits the P-gp and breast cancer-resistant protein transporters, and verapamil inhibits several organic cation transporters in addition to the P-gp transporter (US Department of Health and Human Services, 2006). It is unlikely that this affected the study results because it was determined that naloxegol is not a substrate of other transporters in other assays (2013 AstraZeneca unpublished data).

### Brain Permeation of Naloxegol in a Rat In Situ Brain Perfusion Model

Brain uptake rates for naloxegol, naloxone, and two reference standards with high or low permeability are presented in Table 4. The brain uptake rate of naloxegol was similar to that of atenolol, a low-permeation standard with no appreciable brain uptake, whereas the brain uptake rate of naloxone was similar to that of antipyrine, a high-permeability standard. Relative to naloxone, the brain uptake rate of naloxegol was reduced approximately 15-fold.

**TABLE 4:** Comparative brain permeation of naloxegol in the rat Data are mean ± S.D.

Drug	Brain Uptake
	*pmol/g brain/s*
Naloxegol*^a^*	4.1 ± 1.4
Naloxone*^a^*	60.2 ± 13.7
Antipyrine*^b^**^,^**^c^*	28.2 ± 14.3
Atenolol*^c^**^,^**^d^*	5.2 ± 2.2

^a^ *n* = 3.

^b^ Reference standard with high penetration.

^c^ *n* = 32.

^d^ Reference standard with low penetration.

### Overall Distribution of Naloxegol: Quantitative Whole Body Autoradiography

**Fig. 4.**
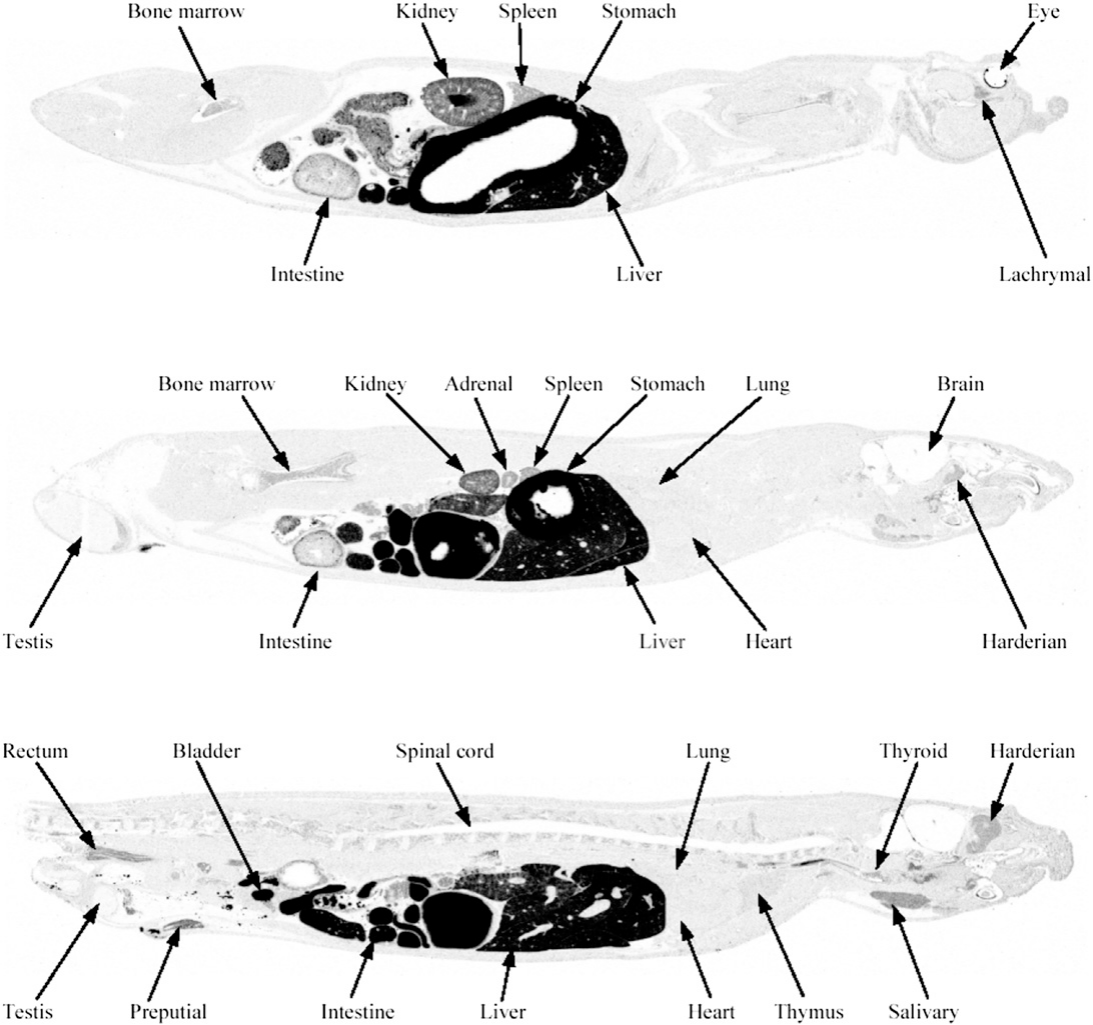
Quantitative whole body autoradiogram of sections through a male pigmented rat at 1 hour after the administration of a single oral dose of [^14^C]naloxegol (50 mg/kg). Note the absence of radioactivity in the brain.

Figure 4 is a representative QWBA profile of the radioactivity distribution in a male rat at 1 hour after administration of a single oral dose of [^14^C]naloxegol at a target dose of 50 mg/kg. The distribution of radioactivity was low or undetectable throughout the brain and spinal cord, particularly relative to its distribution in the blood. Radioactivity levels in the brain and spinal cord were not distinguishable from baseline by 24 hours postdose (Table 5). Total radioactivity was widely distributed to and rapidly eliminated from most tissues (Table 5). The relatively high levels of total radioactivity observed in the liver, kidney, and glandular and pigmented tissues were expected; high distribution in these tissues is typical of many compounds.

**TABLE 5:** Concentrations of radioactivity in selected tissues after administration of [^14^C]naloxegol (50 mg/kg) to adult male and pregnant female rats

Tissues	Concentration (*μ*g Eq/g)					
(M/F)	0.5 h	1 h	4 h	24 h	48 h	168 h
Blood (M)	3.03	4.11	2.14	N.D.	N.D.	N.D.
Blood (F)	12.38	7.80	6.92	N.D.	N.D.	NST
Brain (M)	BLQ	0.21	0.14	N.D.	N.D.	N.D.
Brain (F)	0.34	0.15	0.28	ND..	N.D.	NST
Large intestine wall (M)	8.83	14.09	12.09	N.D.	N.D.	N.D.
Large intestine wall (F)	23.61	25.20	37.45	N.D.	N.D.	NST
Liver (M)	43.40	66.49	58.74	4.61	7.74	1.03
Liver (F)	68.67	56.01	58.78	4.42	2.68	NST
Spinal cord (M)	0.06	0.15	0.02	N.D.	N.D.	N.D.
Spinal cord (F)	0.24	0.10	0.12	N.D.	N.D.	NST
Placenta (F)	21.09	12.89	14.78	0.16	BLQ	NST
Uterus (F)	31.19	82.21	185.03	19.04	4.00	NST
Fetus (whole) (F)	3.10	1.99	2.23	0.17	BLQ	NST

BLQ, below the limit of reliable quantitation; N.D., not distinguishable; NST, no sample taken.

## Discussion

In the ENS, opioid actions at *µ*-opioid receptors inhibit gastrointestinal motility, transit, and secretions. These actions account for the constipating effects seen in a high proportion of patients receiving opioids for pain management (Pappagallo, 2001; Cook et al., 2008; Bell et al., 2009b). In the present investigation, results demonstrate that naloxegol acts antagonistically at *µ*-opioid receptors, both in vitro and in vivo; however, reduced penetration of naloxegol into the CNS in vivo restricts the action of naloxegol to the PNS, thereby targeting the cause of OIC without impacting pain management.

**Fig. 5.**
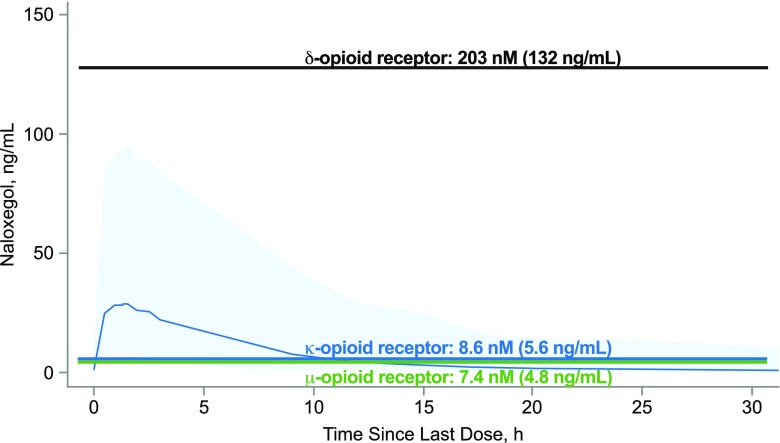
Predicted plasma concentration-time profile for oral naloxegol 25 mg. At this maximum clinically approved dose, naloxegol exposure is predicted to be insufficient to antagonize the *δ*-opioid receptor. The light blue area represents the 90% confidence interval (*N* = 500 simulations from phase 3 studies).

In vitro experiments have shown that naloxegol binds to the human *µ*-opioid receptor with high affinity, acting as a competitive neutral antagonist. Collectively, these data support a selective interaction of naloxegol at the *µ*-opioid receptor. Although naloxegol also appears to bind the *δ*-opioid receptor, simulated exposure based on a population pharmacokinetics model (Al-Huniti et al., 2016) suggests that plasma concentrations resulting from the oral administration of naloxegol at 25 mg (the maximum clinically approved dosage) are unlikely to be high enough to approach the K_i_ of the human *δ*-opioid receptor (Fig. 5). Hence, we expect naloxegol to have no relevant antagonism of *δ*-opioid receptors in vivo at recommended doses in patients with OIC.

Based on the affinity of naloxegol determined at the cloned rat *κ*-opioid receptors (*K*_i_ = 8.65 nM), naloxegol is likely to have significant binding to peripheral *κ*-opioid receptors in humans at therapeutic doses. In addition, in the human *κ*-opioid receptor [^35^S]GTP*γ*S functional assay, naloxegol was found to be a partial agonist at the receptor (EC50 of 45 nM, E_max_ of 39%). Hence, a potential clinical effect of naloxegol at peripheral *κ*-opioid receptors cannot be ruled out. However, as the heterologous overexpression of human *κ*-opioid receptors in the [^35^S]GTP*γ*S functional assay (1700 fmol/mg protein) can amplify low levels of partial agonism, the isolated field-stimulated rabbit vas deferens assay was used to determine whether the partial *κ*-agonism of naloxegol in the [^35^S]GTP*γ*S assay translates to a more physiologic system. In the rabbit vas deferens assay, naloxegol elicited no agonist activity at *κ*-opioid receptors at concentrations up to 10 *µ*M. Thus, the partial agonism of naloxegol at human *κ*-opioid receptors in vitro might not translate to actual *κ*-agonism in humans. Nonetheless, peripheral *κ*-opioid agonists such as asimadoline and fedotozine were found to be safe and well tolerated in clinical trials (Delvaux. 2001; Mangel et al., 2010), and hence agonism of peripheral *κ*-opioid receptors is likely to cause no safety and tolerability concerns. Specific effects related to agonism of central *κ*-opioid receptors such as dysphoria, sedation and increased diuresis were not observed in clinical trials with naloxegol (Chey et al., 2014; Webster et al., 2014; Eldon et al., 2015).

The PAMORAs naloxegol and methylnaltrexone and the centrally acting agent naloxone have a morphinan ring-based structure in common (US National Library of Medicine TOXNET Toxicology Data Network, http://chem.sis.nlm.nih.gov/chemidplus/), whereas alvimopan, a PAMORA approved for the treatment of postoperative ileus, belongs to a different chemical class (Kraft et al., 2010) . The selectivity profile for naloxegol at opioid receptor subtypes in vitro (Table 2) is comparable to that of methylnaltrexone and naloxone (Beattie et al., 2007), with a rank order of affinities of *µ* ≤ *κ* < *δ*. Like naloxone, naloxegol is a neutral antagonist at the human *μ*-opioid receptor, with essentially no direct agonist activity (5.1% at 30 *μ*M in the low *μ*-opioid receptor expression system and 7.8% at 5 *μ*M in the higher expression system). Methylnaltrexone shows a small degree of partial *μ*-agonism in our experiments (14.4%) and 9% to 11% according to a previous report also using [^35^S]GTP*γ*S as the functional assay (Beattie et al., 2007). However, an important difference between PAMORAs and naloxone is that doses of naloxone sufficient to reverse the GI-related effects of opioid agonists in vivo also inhibit the centrally mediated effects of opioids in mice (Zimmerman et al., 1994), rats (Brown et al., 1983; Gmerek et al., 1986), and humans (Sykes, 1996; Liu and Wittbrodt, 2002). This is explained by the ability of naloxone to penetrate the CNS, limiting its utility for patients with OIC. In the context of CNS uptake and potential to reverse the analgesic effect of opioids, the PEG moiety of naloxegol is important because it confers properties that limit its passive permeability through CNS membranes and render it a substrate for the P-gp transporter, thereby limiting its distribution into the CNS (Faassen et al., 2003). Results of the Caco2 transport assay suggest that the PEG moiety of naloxegol reduces its passive permeability compared with naloxone and makes it susceptible to efflux by P-gp.

The P-gp transporter acts as an important gatekeeper into the CNS and is capable of expelling a range of foreign compounds from the CNS (Osherovich, 2009). This same activity can be exploited to restrict the distribution of appropriately designed drugs to the periphery, thereby limiting the potential for CNS effects. P-gp is able to restrict the distribution of naloxegol to the CNS, as demonstrated by results from the brain uptake and QWBA studies in the rat. The CNS distribution of naloxegol when coadministered with a strong P-gp inhibitor has been tested in a drug-drug interaction study with quinidine and was found to be unaffected (Bui et al., 2016). This is because currently marketed drugs that are P-gp inhibitors are not sufficiently potent to inhibit P-gp at the BBB (Kalvass et al., 2013).

Overall, the results of in vivo assessments of the effects of naloxegol on the PNS- and CNS-mediated effects of morphine are consistent with the study results showing a decreased rate of brain uptake for naloxegol compared with naloxone. Naloxegol, a PEGylated derivative of naloxol, which is derived from naloxone, demonstrates a significant reduction in antagonism of CNS opiate receptors (enabling the retention of morphine-induced analgesia) while not abolishing the peripherally mediated receptor antagonist properties of naloxone. Indeed, naloxegol demonstrated the ability to reverse morphine-induced GI transit delay while maintaining a substantial analgesic effect. The latter findings are complemented by brain permeation studies in the rat, which revealed that naloxegol penetrated the CNS 15 times more slowly than naloxone. QWBA analysis supports the findings from the in vitro Caco2 and in vivo brain permeation studies, depicting the poor distribution of drug-derived radioactivity throughout the tissues of the CNS. The in vivo pharmacology studies of naloxegol also support clinical pharmacodynamic data from a single ascending dose study in which naloxegol reduced orocecal transit time in healthy male volunteers who had been administered morphine, demonstrating dose-ordered antagonism of the peripheral effects of morphine while producing no change in morphine-induced miosis, a centrally mediated effect (Eldon et al., 2015).

## Conclusions

The combined results of these studies corroborate information from the phase 3 clinical studies that demonstrated that naloxegol relieves symptoms associated with OIC in patients with chronic noncancer pain via antagonism of the *µ*-opioid receptor in the ENS while preserving opioid-induced analgesia mediated by the CNS (Chey et al., 2014; Webster et al., 2014).

## Abbreviations

ANOVA: analysis of variance
BBB: blood-brain barrier
Caco2: human colon adenocarcinoma cell line
CHO: Chinese hamster ovary
CNS: central nervous system
DAMGO: [d-Ala_2_, *N*-MePhe_4_, Gly-ol]-enkephalin
DPDPE: [d-Pen_2_, d-Pen_5_]-enkephalin
DTT: dithiothreitol
EC_50_: half-maximal effective concentration
ENS: enteric nervous system
E_max_: maximum effect
GI: gastrointestinal
[^35^S]GTP*γ*S: guanosine 5-*O*-(3-[^35^S]thio)triphosphate
HEK: human embryonic kidney
HPLC: high-pressure liquid chromatography
IC_50_: half-maximal inhibitory concentration
*K*_i_: equilibrium dissociation constant for the inhibitor
OIC: opioid-induced constipation
PAMORA: peripherally acting *µ*-opioid receptor antagonist
P_app_: apparent permeability
PEG: polyethylene glycol
P-gp: P-glycoprotein
p*K*i: negative log of the equilibrium dissociation constant for the inhibitor
PNS: peripheral nervous system
QWBA: quantitative whole body autoradiography
U69593: *N*-methyl-2-phenyl-*N*-(7-pyrrolidin-1-yl-1-oxaspiro[4.5]decan-8-yl)acetamide
